# Syncope and Asphyxia

**Published:** 1892-09

**Authors:** G. H. Wilson

**Affiliations:** Cleveland, O.


### Syncope and Asphyxia.

BY G. H. WILSON, D.D.S., CLEVELAND, O.

Read before the Northern Ohio Dental Society, Cleveland, May, 1892.

There are three permanent causes of death or modes by which a human creature ceases to exist. While death may be said to be from any one of these three primary causes it is usually complicated and combines any two, or all three of the phenomena.

The causes are associated with the three chief vital organs of the body; the heart, lungs and brain. Death beginning at the heart is said to be syncope; at the lungs, asphyxia; at the brain, coma.

We may have any one or all of these conditions without death, but when we consider that death must come by one or more of these expressions of dissolution we are the better able to comprehend their significance.

The phenomena known as syncope we will define as failure in the action of the heart, which is speedily followed by symptoms resulting from anæmia of the nerve centres, and these by failing pulmonary functions.

The chief predisposing causes of syncope are, in the female sex, a nervous temperament, weakness, an impoverished condition of the blood and organic structural deficiencies.

The exciting causes are numerous; the more important one, want of sufficient blood in the cavities of the heart, as from excessive hemorrhage or sudden removal of pressure from any of the great blood vessels.

Another cause is an inadequate supply of blood to the cardiac walls, as from obstruction of the coronary arteries; or a supply of impure blood, as in low fevers, or a hot or crowded room.

Another cause is partial or complete paralysis of the muscular tissue of the heart, either from organic changes or from nervous disturbance which may be either centric, reflex, or intrinsic. The last we will name is continued spasmodic contraction of the heart.

Syncope may come on quite suddenly or cause instant death, but usually there are premonitory symptoms before actual insensibility occurs.

The premonitory symptoms are giddiness, trembling, with sinking in the epigastrium ; nausea and sometimes vomiting ; pallor, with drawn features ; chilliness and shivering with clammy perspiration ; a very rapid and weak pulse, though the large arteries may throb ; marked disturbances of the senses of sight and hearing.

Asphyxia we define as suffocation or lack of oxygenation of the blood from whatever cause. To us, as dentists, this condition is far more circumscribed than syncope. The causes are closure of the glottis, larynx or trachea, either from a foreign body or spasmodic action.

Another cause is a lack of free oxygen, whether by forcible means or substituting a gas destitute of or incapable of supplying the necessary element to the lung tissue.

Still another cause is through the nervous system whether centric, reflex or intrinsic. Last, insufficient blood supply.

We will recognize this phenomenon by the two clinical symptoms, impeded or suspended respiration, and the color of the surface of the body, which will range from a slight turgidity of the mucous membranes to a decided blue-black of the skin.

Coma is characterized by a stupor or insensibility which is soon followed by disturbances of respiration and circulation. In practice we are liable to have all of these conditions presented. Syncope is most common and ordinarily the least dangerous. The danger to the patient and our responsibility will depend upon the conditions under which the patient fainted. If the dentist has not administered a drug or anæsthetic he will not be held responsible should death supervene ; but under such conditions there is little danger of a fatal result, though possible.

When the exciting cause is mental, of whatever nature, or from exhaustion, the treatment will be to do what nature always attempts to do, that is, to cause the patient to assume a recumbent position; give free use to the muscles of respiration, apply cold water to the face and chest, volatile stimulants to the nostrils and stimulants to the mouth if the patient is able to receive them. The diffusible ammoniacal preparations are to be preferred. In the domain of anæsthesia we will have these three conditions mostly to contend with; here they are especially associated with danger and demand our intelligent attention.

Dr. Lyman makes this statement in his work on Anæsthesia: "A dentist's first choice should be nitrous oxide; second and last, sulphuric ether; never chloroform." As there are very few who give ether to-day without the presence of a physician we will place this last article with chloroform, as far as this paper is concerned, and confine ourselves to the one remaining general anæsthetic, nitrous oxide gas.

Clinical experience has now demonstrated that the gas is not only the safest but in the hands of an ordinarily intelligent and careful dentist is practically safe. While there are records of a number of deaths associated with nitrous oxide gas Prof. Guilford says he has yet to learn of a single fatal case from the liquid gas. That there is danger in its use is evident from the large number of men who have abandoned it because of the unfavorable symptoms they have observed. And no one with much experience in its administration will have escaped these unfavorable cases.

I believe we are justified in saying that syncope is not the effect of nitrous oxide, but it is generally accepted that it is incidentally present and is dangerous in proportion as the patient is more or less filled with the gas, thns preventing its elimination.

Asphyxia is the one condition we have to guard against in the administration of gas. It is quite generally conceded that gas is a true anæsthetic and produces its effects by direct action upon the nerve centres. That super-oxidation does not take place at all, and that asphyxia is only incidental; but where there is a good respiratory organization and the nervous system is not unfavorably acted upon by outside influences there will be sufficient oxygen retained in the system to supply the wants of nature, and nature will tolerate the presence of the inert gas.

Should the face indicate the presence of an undue amount of carbonic acid from whatever cause, it can generally be quickly corrected by removing the face piece and admitting one, two or more breaths of pure air when the undiluted gas can be admitted again with probably no further trouble.

Should respiration cease entirely and the patient become rapidly black in the face there is certainly no time to be lost. Throw the patient directly forward bringing the thorax upon the knees; this will compress both the abdomen and thorax and largely expel the contents of the lungs.

With a napkin, pliers or tenaculum draw the tongue well forward; with the finger ascertain that the fauces are well opened, then place the body in a recumbent position, observing that the head is so placed as to make the air passages as straight as possible, when, if respirations have not begun give diligent attention to artificial respiration. During this condition all volatiles, water or rubbing the extremities are useless as the nerve terminals are thoroughly anæsthetized and are not capable of responding to such stimulants. What the system *needs and must have*, is *oxygen*.

We are to-day in the midst of a craze for local anæsthesia, the active ingredient of which is cocaine. In this I believe we have a new danger to contend with. While I have not as yet seen anything written scientifically upon its effects on the system we know it has a decided toxic action, and this I believe is directly upon the nerve centres which we know as coma. Circulation and respiration only being affected because of the sedative action upon the brain. The treatment consists of active stimulation, thus assisting nature to diffuse the poison throughout the whole system thereby reducing its potency to such an extent that nature can successfully eliminate the drug.

Trusting that these few thoughts will evoke a discussion and that we shall have the results of your valuable experience and observations, I close.

